# Heavy Metal Content in Thoracic Tissue Samples from Patients with and without NSCLC

**DOI:** 10.1155/2014/853158

**Published:** 2014-07-09

**Authors:** Jessica Q. Tran, Alexandra Dranikov, Anita Iannucci, Walter P. Wagner, Janine LoBello, Jeffrey Allen, Glen J. Weiss

**Affiliations:** ^1^Wellness Integrative, 14795 Jeffrey Road, Suite 101, Irvine, CA 92618, USA; ^2^Arizona State University, 1151 South Forest Avenue, Tempe, AZ 85287, USA; ^3^University of California, Irvine, CA 92697, USA; ^4^The Translational Genomics Research Institute, 445 N 5th Street, Phoenix, AZ 85004, USA; ^5^Humboldt Medical Specialists, Hematology/Oncology, 2504 Harrison Avenue, Suite C, Eureka, CA 95501, USA; ^6^Cancer Treatment Centers of America, 14200 W Celebrate Life Way, Goodyear, AZ 85338, USA

## Abstract

*Objectives*. Environmental factors expose an individual to heavy metals that may stimulate cancer growth preclinically including non-small cell lung cancer (NSCLC) cells. Here, we examine the prevalence of four heavy metals present in postsurgical tissues from individuals with and without NSCLC. * Materials and Methods*. Thoracic tissue samples from two separate sample sets were analyzed for cadmium (Cd), arsenic (As), mercury (Hg), and lead (Pb) content. * Results*. In the first sample set, there was no significant measurable amount of Pb and Hg found in either NSCLC tissue or nonmalignant lung tissue samples. Cd was the most prevalent heavy metal and As was present in moderate amounts. In the second sample set, Cd was measurable across all tissue types taken from 28 NSCLC patients and significantly higher Cd was measurable in noncancer benign lung (*n* = 9). In the NSCLC samples, As was measurable in moderate amounts, while Hg and Pb amounts were negligible. * Conclusion*. Cd and As are present in lung tissues for patients with NSCLC. With existing preclinical evidence of their tumorigenecity, it is plausible that Cd and/or As may have an impact on NSCLC development. Additional studies examining the prevalence and association between smokers and nonsmokers are suggested.

## 1. Introduction

According to the American Cancer Society, approximately 228,200 unique cases of lung cancer will be diagnosed in the United States in 2013 [1]. Additionally, there will be over 159,000 lung cancer deaths in 2013, which accounts for approximately 27% of all cancer related deaths [1]. The majority (85%) of lung cancer cases are non-small cell lung cancer (NSCLC) which includes adenocarcinoma, squamous cell carcinoma, or large cell carcinoma histologic subtypes [2].

Individuals are exposed to toxic heavy metals, specifically cadmium (Cd), arsenic (As), mercury (Hg), and lead (Pb), through both local environmental factors and lifestyle choices—specifically smoking. A statistically significant dose-response relationship has been found between the soil concentration of several heavy metals (including As, Cd, Hg, and Pb) and the rise of different types of lung cancer such as NSCLC and small cell lung cancer (SCLC) [3]. The tobacco plant is efficient at concentrating these heavy metals from soil and groundwater and the average cigarette contains approximately 1-2 *μ*g of Cd, 50–120 ng of As, 2.95–10.2 ng of Hg, and 1-2 mg/kg of Pb [4–6]. Therefore, excessive smoking may lead to high uptake of these toxic heavy metals in lung tissue and stimulate the growth of lung cancer [7].

**Figure 1:**
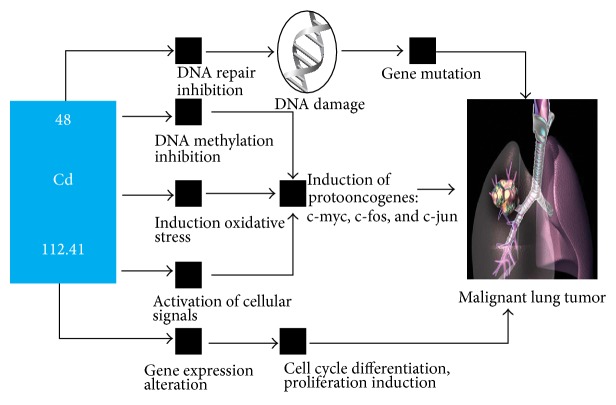
Cd as a carcinogenic agent in cellular pathways [11]. Line with arrow: process proceeds to next step.

**Figure 2:**
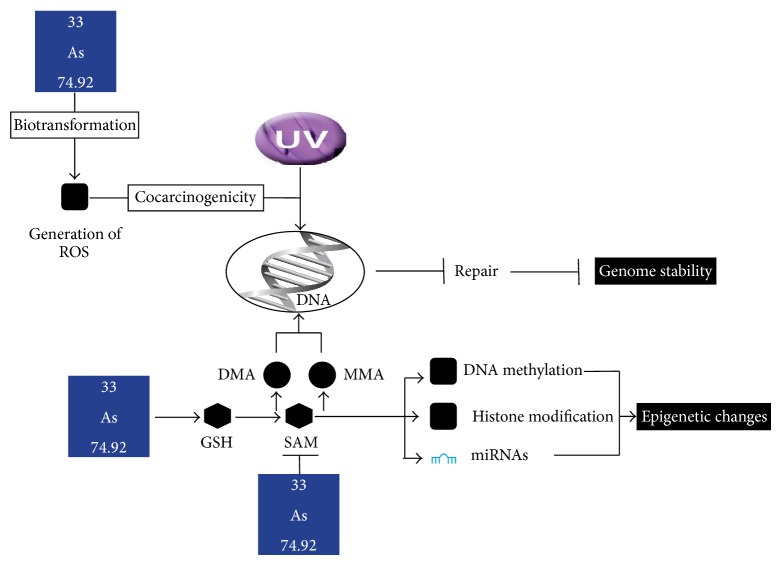
As acting as a carcinogenic agent in cellular pathways [12]. Line with perpendicular T: process inhibited; line with arrow: process proceeds to next step; UV: ultraviolet light; ROS: reactive oxygen species; DMA: dimethylated species; MMA: monomethylated species; GSH: glutathione; SAM: S-adenosylmethionine; miRNAs: microRNA.

Other vehicles of heavy metal uptake into the body, such as ingestion, have also been examined in a study in China and found to be correlated with the onset of lung cancer [8]. Cd and As in particular have been found to be linked to lung cancer due to their prevalence and ubiquity in developed countries (Figures 1 and 2) [9, 10].

Heavy metals lead to gene expression alteration resulting in DNA methylation and/or DNA repair inhibition during the cell cycle synthesis and replication phases. This is especially true for the processes by which Cd and As interact within the nucleus of cells and disrupt proper replication [11, 12]. In particular, chronic Cd exposure in vitro was found to induce a number of oncogenetic changes including increased expression of K-Ras and N-Ras as well as decreased expression of tumor suppression genes p16 and SLC38A3, thereby directly linking Cd to lung carcinogenesis [13]. These genomic changes and mutations may be associated with an increased chance of an individual developing lung cancer at some point in his or her life. In this report, we examined the prevalence and amount of heavy metals present in tissue from individuals with and without NSCLC.

## 2. Materials and Methods

Fifteen patient cases initially treated at the University of Tennessee Health Science Center, Memphis, TN (UT), were matched for stage, histology, gender, age, and race. Formalin-fixed paraffin-embedded (FFPE) tissue samples were retrospectively collected after prior approval under Exemption 4 of Title 45 Code of Federal Regulations (CFR) concerning retrospective study of existing data from treatment-naïve stage I and stage II NSCLC patients who underwent surgical resection. Two 20 micron thick slides derived from FFPE tissues were obtained and tissue was scraped into a code-labeled 1 mL Eppendorf centrifuge tube for analysis.

Twenty-eight histologically confirmed NSCLC (adenocarcinoma, squamous cell carcinoma, or large cell carcinoma) and 9 noncancer benign lung tissue samples from patients initially treated at Maricopa Medical Center, Phoenix, AZ (MMC), were retrospectively collected after prior approval under Exemption 4 of Title 45 CFR concerning retrospective study of existing data. For cancerous lung, areas with ample viable tumor cell concentrations with minimal inflammation were selected. For noncancer patient benign lung, areas were selected from regions of lung comprised of large concentrations of “normal-appearing” pneumocytes and alveoli. The characteristics of “normal-appearing” lung included minimal inflammation, edema, hemorrhage, and areas with smaller nonemphysematous alveolar/air spaces.

UT and MMC hematoxylin and eosin (H&E) slides of the FFPE blocks were reviewed by a pathologist (J.L.) and marked. MMC H&E sections of the FFPE tissue blocks were biopsied with two 1 mm diameter tissue microarray (TMA) cores. Samples were submitted in a code-labeled 1 mL Eppendorf centrifuge tube. Both the UT and MMC deidentified samples were submitted for heavy metal content analysis to Doctor's Data (St. Charles, IL) in a random, nonsequential order. Harnessed tissue was analyzed via inductively coupled plasma mass spectroscopy (ICP-MS) for concentrations (in *μ*g per g of tissue) of Cd, As, Hg, and Pb. Concentrations were measured from primary lung cancer, benign lung tissue adjacent to tumor (benign adjacent lung) and where applicable to malignant lymph node, benign lymph tissue adjacent to tumor involved lymph node (benign adjacent lymph node), benign lymph node, distant nonmalignant lung tissue, and noncancer patient benign lung. Negative controls were paraffin without tissue from UT and MMC FFPE blocks.

For UT, the nonparametric Wilcoxon signed-rank test was used. For MMC, the Wilcoxon signed-rank test was used to make intracase comparisons and the nonparametric Mann-Whitney *U* and Kruskal-Wallis tests were used to examine intercase differences.

## 3. Results

From UT, there were 75 tissue samples with an average sample weight of 2.9 mg taken from 15 unique patients with a median age of 64.7 years. Clinical characteristics of the UT cases are summarized in Table 1, while the heavy metal content of UT cases is summarized in Table 2. For the UT negative controls, the median concentrations of Cd, As, Hg, and Pb were all 0. There was no significant (*n* > 0.001 *μ*g/g) amount of Pb and Hg found in either primary lung tumor tissue or distant benign lung samples. As was present in moderate amounts in primary lung tumor tissue (median: 0.019 *μ*g/g) and distant benign lung tissue (median: 0.025 *μ*g/g). Cd was significantly higher in distant benign lung tissue (median: 0.204 *μ*g/g) than in primary lung tumor tissue samples (*P* = 0.013). Concentrations of Cd and As were higher than Hg and Pb in lung tissues of current/former smokers with NSCLC. There were no significant intercase differences for the heavy metal concentrations in primary lung tumor and stage, histology, gender, and race.

From MMC, there were 79 tissue samples with an average sample weight of 7.2 mg taken from 37 unique patients with a median age of 65.0 years. Clinical characteristics of the MMC cases are summarized in Table 3, while the heavy metal content of MMC cases is summarized in Table 4. For the MMC negative controls, the median concentrations of Cd, As, Hg, and Pb were 0, 0, 0.019, and 0, respectively. Squamous cell carcinoma had significantly higher Cd (median: 0.101 *μ*g/g) compared to adenocarcinoma (median: 0.036 *μ*g/g) (*P* = 0.015). There was significantly higher Cd in primary lung tumor (median: 0.065 *μ*g/g) from 28 patients compared to noncancer patient benign lung tissue from 9 patients (median: 0.029 *μ*g/g) (*P* = 0.023) (Supplementary Table S1 available online at http://dx.doi.org/10.1155/2014/853158). There were no other significant differences between primary tumor and histology for the other three heavy metals. Further, there were no significant intercase differences for the heavy metal concentrations based on age or gender in the MMC data set.

## 4. Discussion

Because of the limitations to the analysis of the clinically annotated UT tissue samples, we chose macroscopic tissue scraping to acquire the region of interest for analysis of the UT samples. While the tissue yield was lower than the core punch biopsy used for analysis on the MMC samples, we observed that heavy metals were detectable using ICP-MS. In the clinically matched samples from UT, Cd is the most prevalent heavy metal. Interestingly, Cd was observed more readily in distant nonmalignant lung tissue than in primary tumor samples. One other study has also reported a statistically significant difference between primary lung cancer Cd and paracancerous/distant nonmalignant lung tissue in patients with lung cancer [14]. A mechanism to explain this finding has not been determined.

After confirmation that heavy metal concentration in negative control paraffin was negligible, we proceeded to analyze larger pieces of tissue using core punch biopsy for analysis applied to the MMC cohort. Since survival and outcome data was not available from MMC samples, the correlative statistical analyses we could apply were less robust than what was applied to the UT sample set. As was also measurable in the MMC tissue samples; however the amount of Hg and Pb detected was negligible compared to both Cd and As. Further research is necessary to establish whether the presence of Cd and As in lung tissue (in our cohorts Cd and As are most probably due to cigarette smoke exposure) is a more significant contributor to mutation and cell cycle disruption in lung tissue than Hg and Pb. Others have reported that As, Cd, chromium (Cr), and nickel (Ni) were found in levels up to 3.4 times higher in malignant tissue than in normal tissue [15]. In another smoking-related disease, a study in a Tunisian population revealed that cumulative smoking is directly correlated with exposure to heavy metals which may predispose smokers to increased risk of developing head and neck cancer—with Cr and Ni being especially volatile agents [15, 16]. Additionally, Pb, Cd, Cr, and Ni were present in high concentrations within the serum and bile of patients with gallbladder carcinoma [17].

Prior published data and our results suggest that heavy metals are likely found in a variety of tissues in the respiratory system that come into direct or close contact with heavy metals absorbed into the body via smoking and/or ingestion. It remains to be seen whether Cd or other heavy metals, either individually or in combination, have a more carcinogenic effect on human tissue.

## Supplementary Material

The quantity of heavy metals analyzed in primary NSCLC tumor compared with non-cancer lung tissue from MMC cases is provided in Supplementary Table S1.

## Figures and Tables

**Table 1:** Clinical characteristics of UT cases (*n* = 15).

Median age (range)	64.7 years (36.4–5.4)
Gender	Number of cases
Male	8
Female	7
Smoking status	
Current	7
Former	8
Ethnicity	
Caucasian	9
African American	6
Histology	
Adenocarcinoma	7
Squamous cell carcinoma	8
Tumor stage	
I	7
II	8

**Table 2:** Heavy metal content in the UT tissue samples.

Heavy metal	Median concentration (range) of metal in primary lung tumor site in *µ*g/g	Median concentration (range) of metal in distant benign lung tissue in *µ*g/g	Median concentration (range) of metal in benign adjacent lung tissue in *µ*g/g	Median concentration (range) of metal in malignant lymph node tissue in *µ*g/g	Median concentration (range) of metal in benign adjacent lymph nodes in *µ*g/g	Median concentration (range) of metal in distant benign lymph nodes in *µ*g/g
Cd	0.0580 (not detectable-0.316)	0.2040 (not detectable-2.500)	0.0830 (not detectable-0.942)	0.0260 (not detectable-0.096)	0.0264 (not detectable-0.1058)	0.0512 (not detectable-0.015)
As	0.0305 (not detectable-0.078)	0.0250 (not detectable-0.070)	0.0230 (not detectable-0.266)	0.0480 (not detectable-0.201)	0.0265 (not detectable-0.0832)	0.0238 (not detectable-0.015)
Hg	0 (not detectable-0.015)	0 (not detectable-0.012)	0 (not detectable)	0 (not detectable)	0 (not detectable)	0 (not detectable-0.015)
Pb	0 (not detectable-0.026)	0 (not detectable)	0 (not detectable-0.101)	0 (not detectable-0.057)	0 (not detectable-0.0585)	0 (not detectable-0.015)

**Table 3:** Clinical characteristics of MMC cases.

NSCLC cases (*n* = 28)	
Median age (range)	69 years (58–83)
Gender	Number of cases
Male	14
Female	14
Histology	
Adenocarcinoma	15
Squamous cell carcinoma	11
Large cell carcinoma	2

Noncancer cases (*n* = 9)	
Median age (range)	35 years (25–62)
Gender	Number of cases
Male	5
Female	4
Diagnosis (reasons for lung surgery)	
Gunshot wound	2
Inflammation	2
Granuloma/fibrosis	2
Hemorrhage	1
Contusion	1
Hemothorax	1

**Table 4:** Heavy metal content in the MMC tissue samples from NSCLC cases.

Heavy metal	Median concentration (range) of metal in primary lung tumor site in *µ*g/g	Median concentration (range) of metal in distant benign lung tissue in *µ*g/g	Median concentration (range) of metal in benign adjacent lung tissue in *µ*g/g	Median concentration (range) of metal in benign lung tissue of noncancer patients in *µ*g/g
Cd	0.0650 (0.01–0.914)	0.118 (0.016–0.561)	0.060 (0.011–0.825)	0.029 (0.011–0.323)
As	0.0305 (0.011–0.093)	0.031 (0.023–0.039)	0.022 (0.014–0.099)	0.016 (0.011–0.027)
Hg	0.036 (0.011–15.3)	0.038 (0.014–0.062)	0.052 (0.014–0.099)	0.051 (0.026–0.065)
Pb	0.0235 (0.012–0.043)	0.016 (0.01–0.028)	0.028 (0.014–0.099)	0.04 (0.013–0.119)
