# Supplementary material for: Heavy Metal Content in Thoracic Tissue Samples from Patients with and without NSCLC
Source: Lung Cancer Int. 2014 Jul 9;2014:853158. doi: 10.1155/2014/853158 (PMC4437387; doi:10.1155/2014/853158)
Supplement: Supplementary file 1 — The quantity of heavy metals analyzed in primary NSCLC tumor compared with non-cancer lung tissue from MMC cases is provided in Supplementary Table S1. [file 853158.f1.pdf]

Supplementary Table S1: Heavy Metal Content of Primary NSCLC tumor vs. Non-cancer Lung Tissue from MMC Cases

Heavy Metal	Median concentration (range) of heavy metal in primary lung tumor site of cancer patients in µg/g n=28	Median concentration (range) of heavy metal in primary lung tumor site of non-cancer patients in µg/g n=9
**Cd***	0.065 (0.01-0.914)	0.029 (0.011-0.323)
**As**	0.031 (Not detectable-0.093)	0.016 (Not detectable-0.027)
**Hg**	0.036 (Not detectable-15.3)	0.051 (Not detectable-0.065)
**Pb**	0.024 (Not detectable-0.043)	0.04 (Not detectable-0.119)

Key to Table:

*- There was significantly higher Cd in primary lung tumor compared to non-cancer patient benign lung tissue (p=0.023)
